# Functional Optical Coherence Tomography Enables *In Vivo* Physiological Assessment of Retinal Rod and Cone Photoreceptors

**DOI:** 10.1038/srep09595

**Published:** 2015-04-22

**Authors:** Qiuxiang Zhang, Rongwen Lu, Benquan Wang, Jeffrey D. Messinger, Christine A. Curcio, Xincheng Yao

**Affiliations:** 1 Department of Biomedical Engineering, University of Alabama at Birmingham, Birmingham, AL 35294; 2 Department of Ophthalmology, University of Alabama at Birmingham, Birmingham, AL 35294; 3 Department of Bioengineering, University of Illinois at Chicago, Chicago, IL 60607

## Abstract

Transient intrinsic optical signal (IOS) changes have been observed in retinal photoreceptors, suggesting a unique biomarker for eye disease detection. However, clinical deployment of IOS imaging is challenging due to unclear IOS sources and limited signal-to-noise ratios (SNRs). Here, by developing high spatiotemporal resolution optical coherence tomography (OCT) and applying an adaptive algorithm for IOS processing, we were able to record robust IOSs from single-pass measurements. Transient IOSs, which might reflect an early stage of light phototransduction, are consistently observed in the photoreceptor outer segment almost immediately (<4 ms) after retinal stimulation. Comparative studies of dark- and light-adapted retinas have demonstrated the feasibility of functional OCT mapping of rod and cone photoreceptors, promising a new method for early disease detection and improved treatment of diseases such as age-related macular degeneration (AMD) and other eye diseases that can cause photoreceptor damage.

Many eye diseases, such as age-related macular degeneration (AMD), retinitis pigmentosa (RP), glaucoma and diabetic retinopathy (DR), can produce retinal damage that is associated with severe vision loss including legal blindness. It is well known that different diseases target different retinal cell types. For instance, it is established that rods are more vulnerable than cones in early AMD^1^,^2^. Currently, there is no established strategy to allow objective assessment of localized rod dysfunction at high resolution. In principle, physiological abnormalities in diseased cells can occur before neuron loss and corresponding retinal thickness changes may be detectable. Therefore, functional evaluation of the physiological integrity of retinal cells is important for early disease detection and reliable treatment management. Psychophysical methods, such as Amsler grid test, visual acuity^3^,^4^ and hyperacuity perimetry^5^, are practical in clinical applications, but they involve extensively higher order cortical processing. Therefore, they do not provide exclusive information on retinal function and they lack sensitivity in early disease detection. Electrophysiological methods, such as focal electroretinography (ERG)^6^,^7^,^8^,^9^,^10^, multifocal ERG^11^,^12^ and dark adaptometry^13^,^14^,^15^, allow objective assessment of retinal function. However, the relationship between low spatial resolution in ERG and localized morphological changes is complicated.

Fast intrinsic optical signal (IOS) imaging is a promising alternative to ERG for objective measurement of retinal physiological functions^16^,^17^. Because functional IOS images are constructed through computer-based dynamic differentiation of retinal images recorded at pre- and post-stimulus periods^18^, concurrent structural and functional assessment can be naturally achieved at high resolution. Conventional fundus cameras^19^,^20^,^21^,^22^,^23^,^24^ and adaptive optics^25^,^26^,^27^ have been explored for IOS imaging. However, reliable mapping of fast IOSs that have time courses comparable to retinal electrophysiological kinetics is still challenging^25^, and unclear IOS sources make it challenging for practical applications. Dynamic imaging of living retinal slices has revealed multiple IOS sources in the outer and inner retinal layers. Given its excellent axial resolution to separate signals in different retinal layers, functional OCT has been used to dissect IOS courses in intact retinas of frogs^28^, rats^29^, rabbits^30^, chickens^31^,^32^, macaque^33^ as well as human^34^ retinas. Yao et al. employed a time-domain OCT (TD-OCT) to detect IOS changes in photoreceptor and ganglion layers in isolated frog retinas^28^. Bizheva et al. used TD-OCT to reveal IOS changes in photoreceptor inner/outer segments and plexiform layers in isolated rabbit retinas^30^,^32^. Srinivasan et al. demonstrated for the first time *in vivo* spectral-domain OCT (SD-OCT) imaging of IOSs in rat retinas^29^. They recorded continuously positive IOS changes within 5 s at a frame rate of 6 Hz. However, recent functional OCT study of the macaque retina^33^ revealed both positive signals in the outer segment (OS) and negative signals in the inner segment ellipsoid (ISe). In addition, relatively slow IOS signals were observed in the inner retina. Functional OCT imaging of stimulus-induced IOSs in human retinas has been also demonstrated^34^. Although all these studies reported convincing IOSs, the signal polarities and time courses were not consistent among different experiments. Moreover, averaging of multiple measurements was typically required to ensure a reliable signal to noise ratio (SNR), which limited its application for clinical applications due to inevitable eye movements during long-term measurements. Because of the limited SNR, reliable mapping of fast IOSs that have time courses comparable to retinal electrophysiological kinetics is still challenging^25^.

Using rapid line-scan confocal imaging, we recently demonstrated single-pass *in vivo* IOS imaging of frog retinas, without the requirement of averaging multiple measurements^16^,^35^. Comparative IOS and ERG experiments revealed a close correlation between confocal IOS and retinal ERG a-wave, which has been widely used to evaluate photoreceptor function. The photoreceptor mosaic we observed with the confocal imager is known to reflect the waveguide properties of the OS. Therefore, we speculate that confocal IOSs might be mainly from photoreceptor OSs. However, due to the limited axial resolution of the confocal system, it has been challenging to accurately verify the axial location of the IOS source in the retina, which consists of multiple layers. In order to achieve subcellular identification of IOS sources, we employed a custom-designed OCT with sub-cellular spatial resolution in three dimensions (3.0 μm × 3.0 μm × 3.0 μm) and millisecond temporal resolution (500 Hz). An adaptive algorithm was developed to achieve a high SNR in a single-pass IOS recording. An animal model (*Rana pipiens*), which has rod and cone photoreceptors stratified into different depths (Fig. 1), was selected for this study^36^. As shown in Fig. 1, cone OSs are almost at the level of the ISe of red rods. Transient IOSs, which might reflect an early stage of light phototransduction, are consistently observed at photoreceptor OSs almost immediately (<4 ms) after retinal stimulation. Comparative studies of dark- and light-adapted retinas demonstrate the feasibility of functional OCT mapping of rod and cone photoreceptors.

## Results

### High resolution OCT guided by a combined fundus camera

The custom designed SD-OCT system, equipped with a retinal stimulator and fundus camera, was employed for functional IOS imaging of anesthetized frogs. The integrated fundus camera can provide real time *enface* mapping (Fig. 2a) to guide the transverse location of OCT B-scan recording (Fig. 2b). As shown in Fig. 2a, the blood vessels around the optic nerve head (ONH) can be clearly observed, thus easily enabling localization of OCT B-scan imaging required for IOS measurement. Fig. 2b illustrates an OCT B-scan image across the ONH. Fig. 2c shows comparative OCT and histological images of the frog retina with individual layers labeled^37^. The custom-designed OCT provided sub-cellular resolution to separate photoreceptor outer/inner segments from other retinal layers and the supporting tissues, such as the choroid and sclera (Fig. 2c).

### Spatiotemporal characteristics of fast IOSs

**Figure 1:**
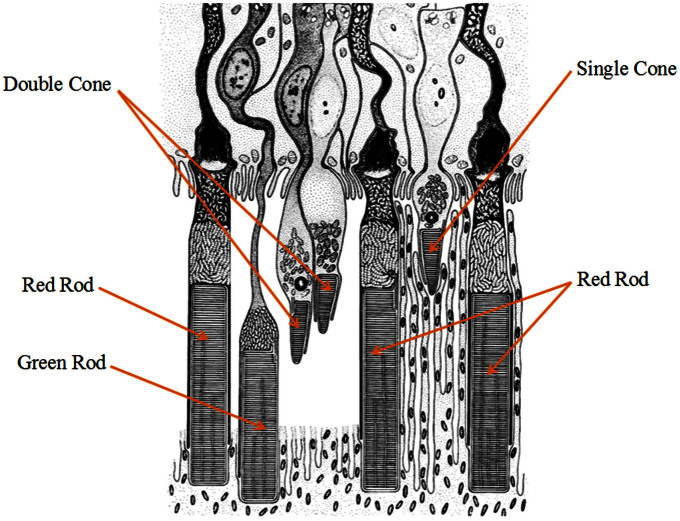
Schematic drawing of retinal photoreceptors of the leopard frog (*Rana pipiens*). Reprinted from *J Ultrastruct Res*, **10**. Nilsson SE. An Electron Microscopic Classification of the Retinal Receptors of the Leopard Frog (*Rana pipiens*). p390-p416, 1964, with permission form Elsevier.

Fig. 3 shows spatiotemporal characterization of functional OCT-IOS imaging with a 10 ms flash stimulus. Figs. 3a1–a3 shows IOS images from both control (without stimulation, Fig. 3a1) and experimental groups (Figs. 3a2 and 3a3, supplementary Movies 1 and 2). These two stimulus-evoked IOS measurements (Figs. 3a2 and 3a3) were recorded from the same retinal area to verify the reproducibility of the IOSs. There were no detectable fast IOSs in the control group, although slow IOSs, which might reflect hemodynamic change or eye movement, were observed after ~2.5 s of the stimulus onset. On the contrary, robust IOSs were constantly observed in experimental groups with fast IOSs predominantly distributed at the outer retina. Quantitative IOS statistics of activated pixel numbers also revealed clear differences between control and experimental groups (Fig. 3b). Combined IOS maps (functional image) and OCT B-scan images (structural image) revealed the photoreceptor OS as the major IOS source in retinal photoreceptors, although slight IOSs were occasionally observed at the photoreceptor ISe and RPE-Bruch's membrane complex (Fig. 3e). We speculate that the IOSs observed at the ISe and RPE bands might be attributed to, or at least be partially affected by, cone and rod OSs, respectively. In order to better understand the anatomic sources of these IOSs around the photoreceptor layer, comparative OCT-IOS and histological images of the outer retina are shown in Fig. 3f. Retinal cone photoreceptors in the histological image are highlighted with green and red dotted circles. As shown in Fig. 3f, the OSs of some cones are located at the level of the rod ISe section, while the OSs of some rods penetrated further toward the sclera into the RPE, which is consistent with the observation in Fig. 1^36^. Therefore, it is reasonable to occasionally observe IOSs at the ISe and RPE layers, even if the OS is the primary IOS signal source in the outer retina. However, the observed IOSs at ISe may also originate from changes at the inner and outer segment boundary during phototransduction.

As shown in Figs. 3a and 3e, both positive and negative IOSs are consistently observed in adjacent retinal areas, which further confirmed that high resolution is essential for functional IOS imaging. Without the necessary spatial resolution to separate localized positive and negative IOSs, IOS sensitivity will be degraded due to integral effects of IOSs with opposite polarities^38^. Given the excellent OCT resolution in both transverse (3 μm) and axial (3 μm) directions, high sensitivity is ensured to achieve robust IOS recording from single-pass measurements (Fig. 3a2 and Fig. 3a3), without an averaging requirement for multiple trials. In order to demonstrate the repeatability of the IOS recording, Fig. 3c illustrates averaged positive and negative IOSs of 6 recording trials from the same retina with identical stimulus parameters. Both positive and negative IOSs occurred within 10 ms, with similar time courses (Fig. 3d). Since the OCT frame speed was set at 100 Hz with a frame size of 140 pixels (lateral) × 200 pixels (axial), for the experiment represented in Fig. 3, it was not possible to measure the actual onset time, which was obviously within the 10 ms interval. In order to characterize the onset time of the observed IOSs, the OCT frame speed was increased to 500 Hz, with a reduced frame size (40 pixels × 200 pixels) for the experiment represented in Fig. 4. With 2 ms temporal resolution, the IOSs were unambiguously detected as early as at 4 ms (Fig. 4c). The overall time courses were similar between 100 Hz (Fig. 3b) and 500 Hz (Fig. 4b) IOSs. However, the SNR of the 100 Hz (20 kHz line rate) IOSs was slightly better than that of 500 Hz (32 kHz line rate) IOSs because the increased line rate resulted in a shorter exposure time and thus a reduced SNR.

In order to further characterize the time courses of positive and negative IOSs, Figs. 4d–4e show representative IOSs of individual pixels. It was observed that localized IOSs can have different onset times. Some of these IOSs were very fast (Figs. 4d1, 4d3, 4d5, 4e1 & 4e3), while others were relatively slow (Figs. 4d2, 4d4, 4d6 & 4e2). The overall temporal curves were also variable. Some of them were sustained at the peak value (Figs. 4d1, 4d3, 4e1 and 4e2), while others gradually recovered to baseline (Figs. 4d5 & 4d6). Figs. 4d1–4d2 represent the pixels from the same axial depth (*y* = 107), however, at adjacent pixels, different time courses were observed. The IOS at the position (*x* = 31, *y* = 107) increased rapidly upon stimulation and reached the peak within 200 ms, while the IOS at position (*x* = 32, *y* = 107) increased almost linearly after the stimulus.

With short flash (10 ms) stimulation, transient IOSs were predominantly observed in the outer retina, although slow IOSs were sparsely observed in the inner retina (Fig. 5a1, supplementary Movie 3). In contrast, prolonged (500 ms) stimulation evoked robust IOSs in both the outer and inner retinal layers (Fig. 5a2, supplementary Movie 4). As shown in Figs. 5a1, 5a2, 5b1–b3 and 5c1–c3, early phase IOSs were consistently confined to the outer retina; i.e., photoreceptor OS. After 1–2 s, later phase IOSs spread into the inner retina (Figs. 5a2 & 5c3) with 500 ms prolonged stimulation. In order to quantify temporal properties of these IOSs in different retinal layers, averaged positive and negative signals from individual layers were illustrated in Figs. 5d1–d5 (10 ms stimulation) and Figs. 5e1–e5 (500 ms stimulation), respectively. As shown in Fig. 5e, with prolonged stimulation, the IOSs of photoreceptor OSs occurred immediately after the stimulus onset, underwent sharp changes first and then linear changes till reached a plateau (Fig. 5e4). However, the onset time of IOSs in other layers, such as the IPL and OPL, was delayed to ~1.5 s (Figs. 5e1–e2). With 10 ms flash stimulation, the IOSs of outer retina increased/decreased sharply and then sustained at their peak values (Figs. 5d4–d5). Compared with the IOSs from outer retinas, the IOSs of inner retina showed relatively slow time courses (Figs. 5d1–d2).

### Comparative IOS imaging of rod and cone photoreceptors

In order to demonstrate the potential of functional IOS imaging of rod and cone functions, comparative experiments were conducted with variable background light controls. In the dark condition, transient IOSs were observed in both cone and rod OSs (Figs. 6a1–6a3, supplementary Movie 5). Combined light reflectance (OCT B-scans) and IOS distribution curves revealed similar signal densities across the photoreceptor OSs and slight IOSs at the ISe and RPE-Bruch's membrane complex regions. In contrast, under the light condition (~200 cd·s/m^2^), the IOS distribution pattern was significantly changed. It was observed that major signals are confined around the cone OS area (Figs. 6b1–6b3, supplementary Movie 6). The light intensity profiles of the outer retina were also changed dramatically (Fig. 6) due to melanosome translocation^39^. The IOS differences under dark and light conditions demonstrated the potential of functional IOS mapping of rod and cone systems, and provided additional evidence to support that the transient IOSs in retinal photoreceptors might be attributed to the OS; i.e., the center of phototransduction.

## Discussion

**Figure 2:**
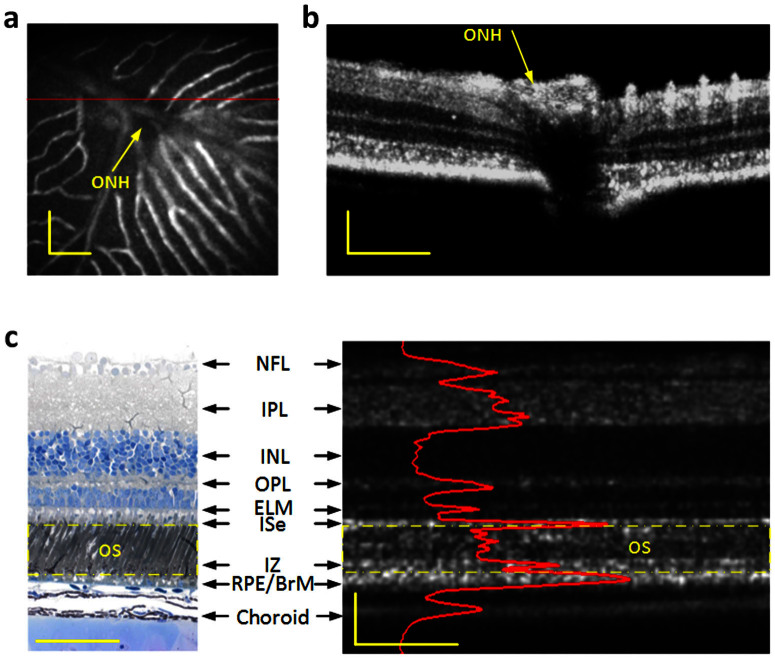
Combined OCT and fundus imaging (a) Fundus image revealed blood vessels around the optical nerve head (ONH). (b) OCT B-scan image of the area marked by the red line in A. (c) Histological image of the frog retina (left) and the corresponding OCT B-scan image with individual layers labeled (right). Outer segment region is marked in yellow dashed rectangles in both the histological image and OCT B-scan image. The OCT B-scan image contains both hyperreflective and hyporeflective bands. The B-scan image is displayed in a linear scale. NFL: nerve fiber layer, IPL: inner plexiform layer, INL: inner nuclear layer, OPL: outer plexiform layer, ELM: external limiting membrane, ISe: inner segment ellipsoid, IZ: interdigitation zone, RPE/BrM: retinal pigment epithelium/Bruch's membrane. Scale bars indicate 100 μm.

**Figure 3:**
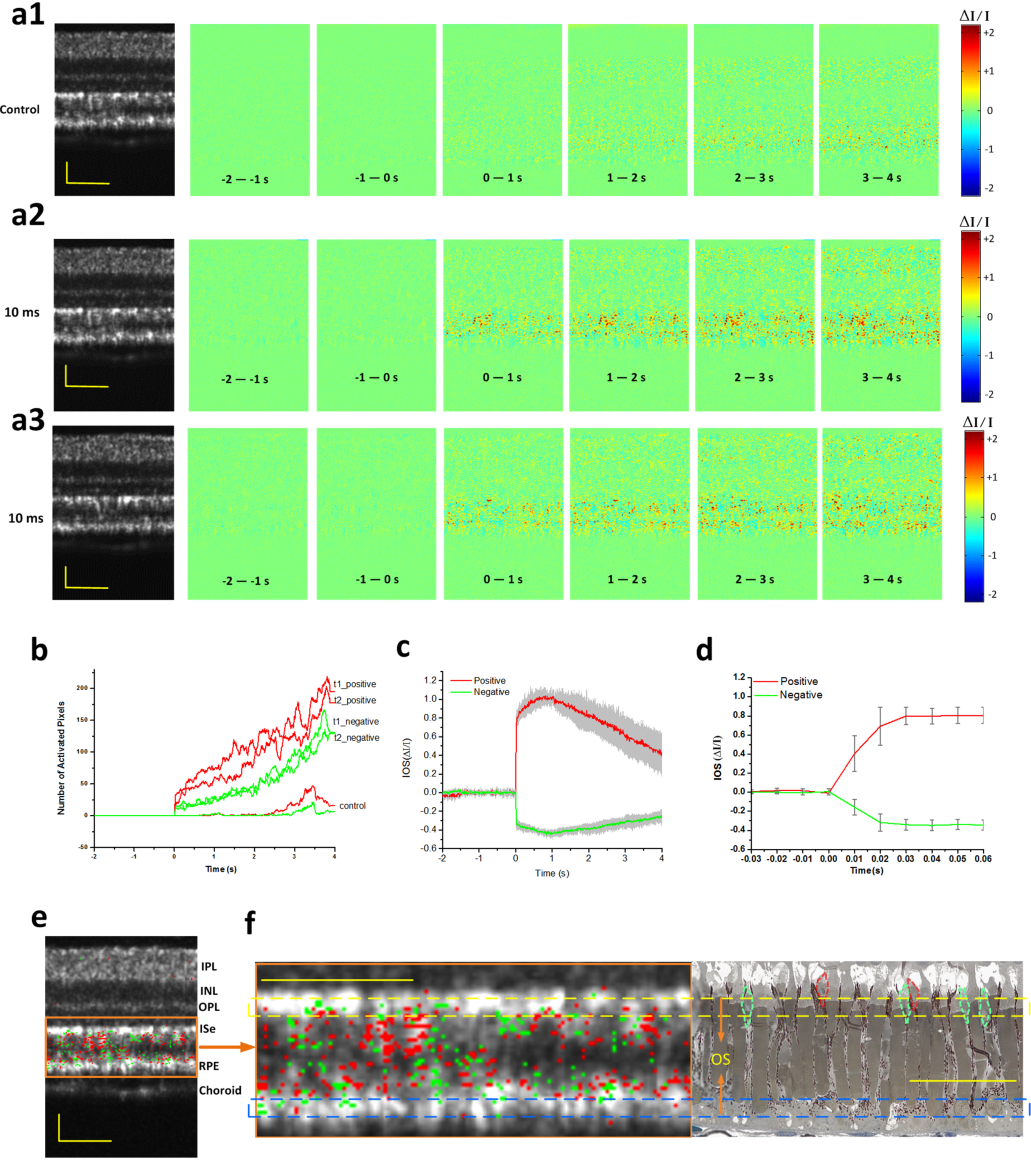
Spatiotemporal characterization of functional OCT-IOS imaging with a 10 ms flash stimulus. Raw OCT images were collected with a frame rate of 100 Hz. Stimulus onset is indicated by time “0”. OCT B-scan images are presented with a linear scale. (a1–a3) OCT B-scan images and spatial IOS image sequences of one control and two experimental groups. All the images were averaged over 10 frames (100 ms interval). Images consisted of 140 pixels (lateral) × 200 pixels (axial), corresponding to 200 μm (lateral) × 360 μm (axial). (b) Temporal curves of the number of activated (positive and negative) pixels corresponding to (a1–a3). (c) Temporal curves of positive and negative IOSs averaged from 6 recording trials. (d) To better visualize the signal onset time, an enlarged profile of the early 80 ms period from c is illustrated. (e) IOS distribution map superimposed on the OCT B-scan image. Positive signals (increasing reflectance) and negative signals (decreasing reflectance) are presented in red and green, respectively. Signal magnitude is not indicated in the image. (f) Comparative OCT-IOS and histological images of the outer retina. In the histological image, cone photoreceptors are highlighted in green or red to show cell sizes and locations. Cone photoreceptor OSs highlighted with red circles are located at the level of the rod ISe. Scale bars indicate 50 μm.

**Figure 4:**
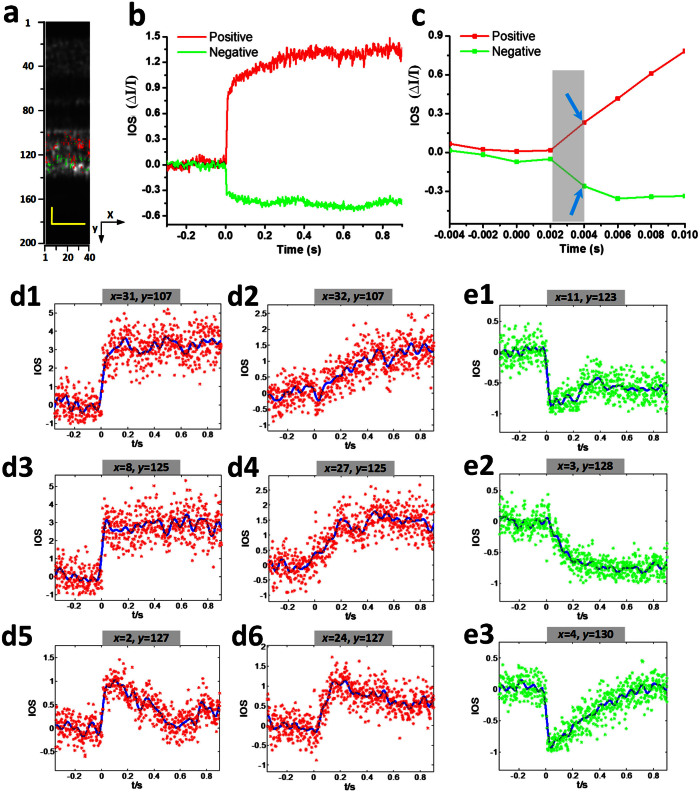
Spatiotemporal characteristics of fast IOSs with a 10 ms flash stimulus. Raw OCT images were collected with a frame rate of 500 Hz. Stimulus onset is indicated by time “0”. (a) The OCT B-scan image consisted of 40 pixels (lateral) × 200 pixels (axial), corresponding to 60 μm (lateral) × 360 μm (axial). Illustrated OCT B-scan images are displayed with a linear scale. The IOS distribution map is superimposed on the OCT B-scan image. (b) Temporal curves of the averaged positive and negative IOSs. (c) To better visualize the IOS onset times, an enlarged profile of the early 14 ms period is illustrated. (d1–d6) Positive IOSs of individual pixels; both raw data (labeled with a star in red) and fitted curves (in blue) are shown. d1 (*x* = 31, *y* = 107) and d2 (*x* = 32, *y* = 107) were selected from the same axial location at adjacent locations in a lateral direction. d3 (*x* = 8, *y* = 125) and d4 (*x* = 27, *y* = 125) share the same axial location but with different lateral positions. d5 (*x* = 2, *y* = 127) and d6 (*x* = 24, *y* = 127) also share the same axial location but with different lateral positions. (e1–e3) Negative IOSs of individual pixels; both raw data (labeled with a star in green) and fitted curve (in blue) are shown. e1 (*x* = 11, *y* = 123), e2(*x* = 3, *y* = 128) and e3 (*x* = 4, *y* = 130) are from different locations. Scale bars indicate 25 μm.

**Figure 5:**
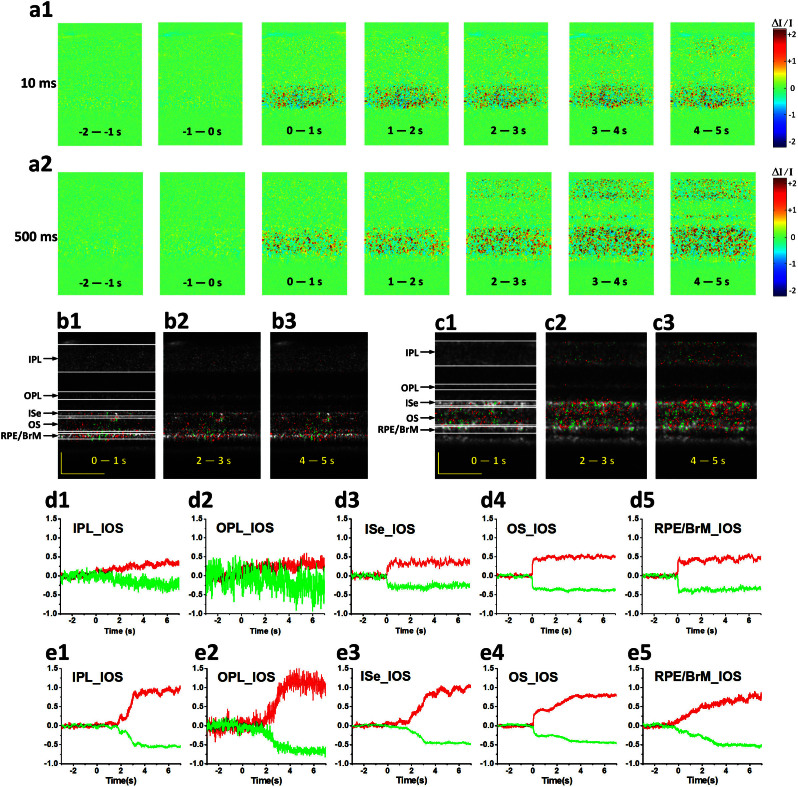
Spatiotemporal characterization of functional OCT-IOS imaging with prolonged recording times. OCT B-scan images were collected with a frame rate of 100 Hz. (a1–a2) Spatial IOS image sequence averaged over 100 frames (1,000 ms interval) with 10 ms stimulation and 500 ms stimulation. OCT B-scan image and OCT-IOS images at 0–1 s, 2–3 s and 4–5 s with 10 ms stimulation (b1–b3) and 500 ms stimulation (c1–c3). The corresponding individual temporal curves of averaged positive and negative IOSs from the IPL, OPL, ISe, OS and RPE/BrM with 10 ms stimulation (d1–d5) and 500 ms stimulation (e1–e5). Scale bars indicate 50 μm.

**Figure 6:**
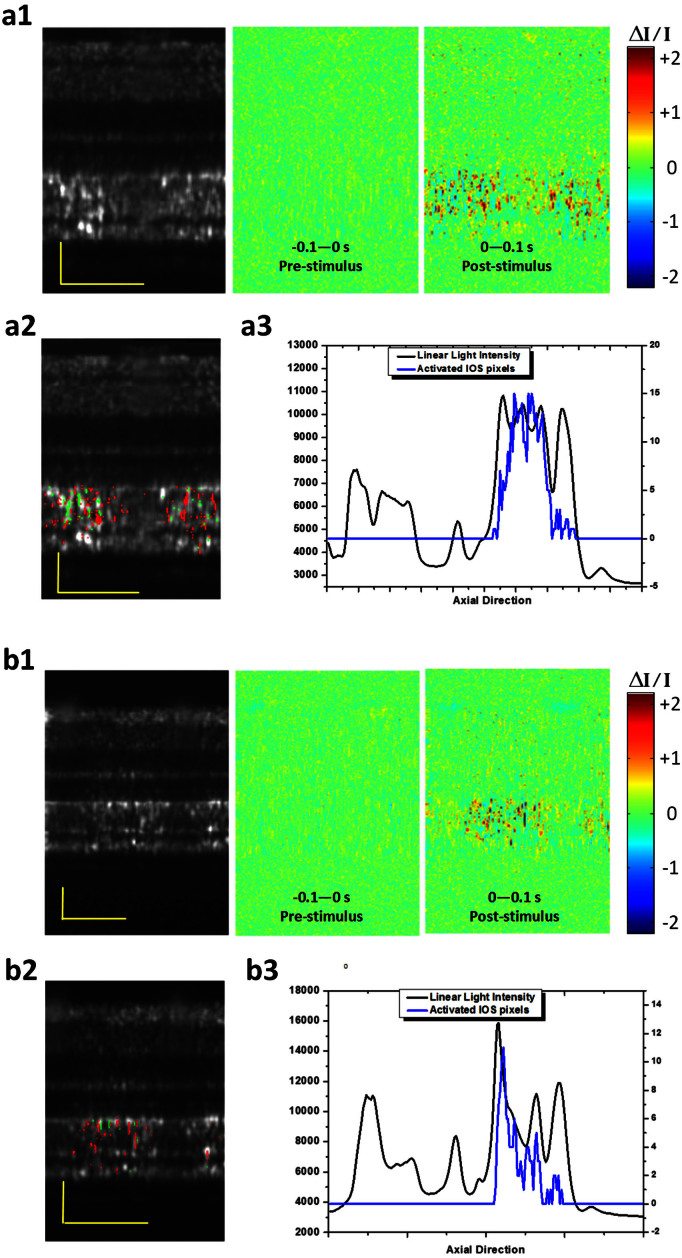
Spatial characteristics of the IOSs in different background light conditions. OCT B-scan images are displayed in a linear scale. (a1) OCT B-scan image and spatial IOS images were collected in a dark condition. Pre-stimulus and post-stimulus images were averaged over 10 frames (100 ms interval) (a2) OCT-IOS image in the dark condition. (a3) Corresponding light intensity and IOS distribution curve across the retina. The light intensity curve was obtained by averaging the light intensity of the OCT B-scan image laterally. The IOS distribution was obtained by calculating the pixel number of IOSs laterally in the axial direction. The signal polarities were ignored for calculating IOS quantities. (b1) OCT B-scan image and spatial IOS images were collected under a light condition (~200 cd·s/m^2^). Pre-stimulus and post-stimulus images were averaged over 10 frames (100 ms interval) (b2) OCT-IOS image in the light condition. (b3) Corresponding light intensity and IOS distribution curve across the retina. Scale bars indicate 50 μm.

In summary, a custom-designed SD-OCT system with a fundus monitor and retinal stimulator was employed to achieve *in vivo* structural and functional imaging of the frog retina at a subcellular resolution. Unprecedented temporal resolution was achieved at 2 ms to reveal robust IOSs as early as 4 ms after retinal stimulation. The high spatiotemporal resolution instrument and adaptive data processing algorithm ensured high SNRs of single-pass measurements, and thus allowed robust IOS imaging without the requirement of averaging multiple experiments. Combined OCT-IOS (structural-functional) images (Figs. 3b, 4a, 5a2 & 5b2) allowed precise identification of the anatomic sources of IOSs. With visible flash (10 ms) stimulation, fast IOSs were predominantly confined to the outer retina, particularly in the photoreceptor OSs. We speculate that the IOSs observed at the ISe and RPE bands might be attributed to, or at least be partially affected by, cone and rod OSs, respectively. As illustrated in Fig. 1, a previous study by Nilsson SE^36^ on the retinal photoreceptors of the leopard frog retina (*Rana Pipiens*), the same type of frog used in our study, clearly showed four types of photoreceptors (i.e., red rod, green rod, single cone and double cone). More than half of the photoreceptors were red rods, agreeing with the histological image in Fig. 2c. They lined up well with each other and their ISe might form the second band while the OS tip and apical processes of the RPE formed the interdigitation zone (IZ) in the OCT B-scan image. The green rods took up only 8% of the total number of photoreceptors and they were often somewhat longer than red rods. Therefore, their OSs penetrated further into the RPE, which probably contributed to the fast IOSs observed in the RPE complex region. For cone photoreceptors, their OS length was much shorter than for rods. Single cones, making up approximately 20% of the total photoreceptors, were shortest, and their OS ended almost at the level of the ISe of red rods. This indicated that the fast IOSs in the ISs band may originate from the OS of single cones. The observed IOSs at ISs may also originate from changes at the inner and outer segment boundary during phototransduction.

Our previous *in vivo* IOS results in the frog retina using a line-scan confocal system showed different time courses under different stimulus light strengths^16^. With stronger light stimulation, the overall IOSs occurred and reached a peak faster. At the same time, it maintained the peak value for several seconds. With weak stimulation, the signals returned to baseline within 1 s^16^. In this study, quantitative analysis of fast IOSs from individual pixels (subcellular scale) revealed varied time courses and different signal polarities (Fig. 4). Positive and negative signals were mixed together, with both containing fast and slow components. One possible reason is there may be localized differences in light absorption efficiency. Although the stimulus light was optimized for uniform illumination, it is impossible to ensure equal light absorption for individual photoreceptors due to the complexity of photoreceptor lengths, locations and spectral sensitivities. Therefore, the light stimulus strengths were probably different for photoreceptors at different pixel positions. This contributed to the signals having differing time courses at the photoreceptor layer.

There are several explanations for the IOSs having different polarities (positive and negative) mixed together. Previous studies with isolated photoreceptor OSs and isolated retinas have demonstrated transient IOSs associated with phototransduction^40^,^41^,^42^. Both binding and release of G-proteins to photoexcited rhodopsin might contribute to the positive (increasing) and negative (decreasing) IOSs^41^. Localized biochemical processes might also produce non-homogeneous light intensity changes; i.e. positive and negative signals mixed together. Moreover, transient photoreceptor movements have been observed due to possible oblique stimulation of a localized area. This might be a key factor contributing to differences in IOS polarity. Supplementary Movie 7 includes two OCT images before and after stimulation to show transient photoreceptor displacement. In this movie, one can see that the relative “bright spots” (outer segments) moving to their neighboring areas make the light intensities of some pixels increase and some pixels decrease. The details of transient photoreceptor displacement has been discussed in our recent publications^43^,^44^.

Slow and low magnitude IOSs were observed in the inner retina with the short flash (10 ms) stimulation. However, further investigation is required to exclude possible artifacts, such as possible hemodynamic change and eye movement. In contrast, robust IOSs were detected in the inner retina activated by the prolonged (500 ms) stimulation, with a delayed time course (1.5–2 s). The delayed IOS response in the inner retina is consistent with our early study of living retinal tissue^45^. We speculate that the observed IOSs in the inner retina may involve complications of nonlinear information processing and retinal adaptation mechanisms. Further investigation is required to achieve an in depth understanding of the IOS sources and mechanisms in the inner retina, which may lead to a new method to advance the study and diagnosis of glaucoma and other eye diseases that may affect inner retinal function.

Comparative study of dark- and light-adapted retinas demonstrated the feasibility of functional OCT mapping of rod and cone photoreceptors, promising a new approach to advance the study and diagnosis of eye diseases that can cause photoreceptor dysfunction. Currently, there is no outright cure for retinal degenerative diseases that produce irreversible damage to photoreceptors and the RPE, of which age-related macular degeneration (AMD) is the most prevalent. A key strategy for preventing vision loss due to late AMD is to preserve function and be vigilant for changes in early AMD that signal progression. Although it is established that rods are more vulnerable than cones in early AMD^1^,^2^, there is no established strategy to provide reliable mapping of rod and cone functions at high resolution. We anticipate that further development of functional OCT-IOS will pave the way toward quantitative assessment of rod and cone photoreceptors with unprecedented accuracy.

## Methods

### Animal preparation

Adult northern leopard frogs (*Rana Pipiens*) were used in this study. Before near infrared (NIR) OCT imaging, frogs were anesthetized through the skin by immersion in tricaine methanesulfonate (TMS, MS-222) solution (500 mg/liter)^46^. After confirmation of anesthesia, the pupils were fully dilated with topical atropine (0.5%) and phenylephrine (2.5%). Subsequently, the frog was placed in a custom-built holder for OCT imaging. The holder provided six degrees of freedom to facilitate adjustment of body orientation and retinal area for OCT imaging^16^. The experimental procedure has been approved by the Institutional Animal Care and Use Committee of the University of Alabama at Birmingham and carried out in accordance with the guidelines of the ARVO Statement for the Use of Animals in Ophthalmic and Vision Research.

### Data Analysis

The raw OCT B-scan images were reconstructed with a linear scale. Raw B-scan images were registered to compensate for eye movements before IOS calculations. The registration was composed of three steps. First, we used the method of principle component analysis (PCA) to evaluate the rotation of retinal layers to compensate for the torsional movement of the eye^34^. Before applying PCA, discrete curve evolution (DCE)^47^ was employed to find a threshold value to separate hyper-reflective signals from the background. Implementation of PCA on two-dimensional coordinates of those hyper-reflective signals yielded two orthogonal components, one denoting the direction parallel to the retinal layers, while the other was perpendicular to the retinal layers. Therefore, we could compensate for the rotational movement after the rotation angle of the retinal layers was known. The second step was to apply localized normalized cross correlation (NCC) to correct for large scale displacements. Dynamic blood vessel movements can corrupt the accuracy of the NCC method. Therefore, we only chose a small localized area without contamination of large blood vessels for estimating image displacement through NCC. The NCC method can only estimate integral displacements. Therefore, we used centroids of hyper-reflective landmarks to compensate for fine (i.e., sub-pixel) displacements, which was the third step of image registration. Because of the high lateral resolution of our system, the second hyper-reflective OCT band at the outer retina became granular rather than continuous, which enabled us to select a small area containing only one hyper-reflective granule. After the granule was segmented from the background using histogram-based methods, the centroid of this granule over time could be obtained, which allowed registration of images with sub-pixel accuracy. Movies showing the images before and after registration can be assessed online (supplementary Movies 8 and 9).

**Figure 7:**
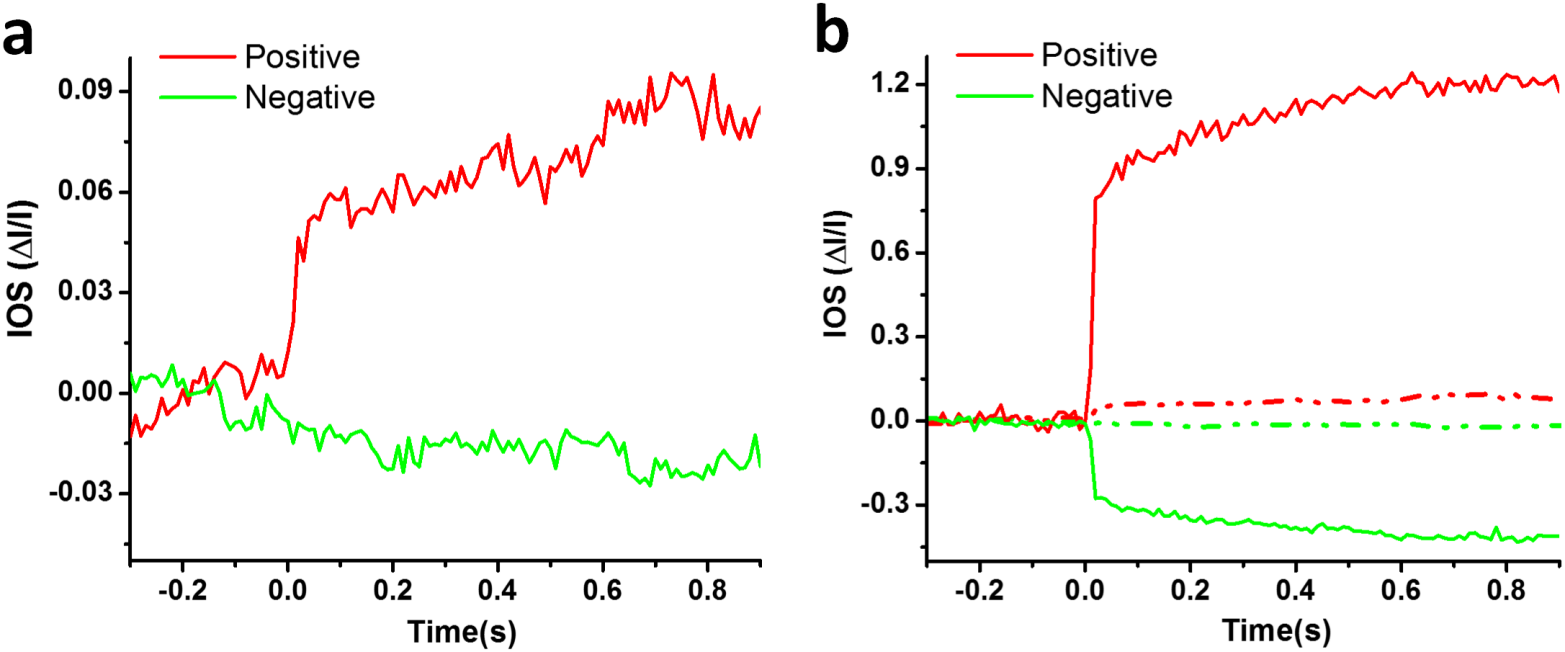
Comparative IOSs before and after applying the adaptive algorithm. (a) Averaged positive and negative IOSs were processed with a traditional method; i.e. for each pixel, if ΔI/I was greater in magnitude than 0.05, and then the pixel was counted as activated. (b) Combined IOSs processed with the adaptive algorithm (solid lines) and those processed with traditional method (dotted lines).

The basic procedures of IOS data processing are described as follows: a high threshold was used to define the stimulus-evoked IOS in the retinal area. The pixel (*x*, *y*) was a negative signal pixel if its light intensity was less than the mean minus three standard deviations five times continuously,
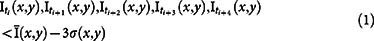
Similarly, the pixel (x, y) was a positive signal pixel if its light intensity was greater than the mean plus three standard deviations five times continuously,
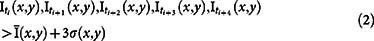
The time interval between *t_i_* and *t_i_*_+1_ was 10 ms for a 100 Hz frame rate. In this way, we could generate an IOS distribution map with either positive or negative signals. The averaged IOSs were calculated based on selected signals rather than the whole area, which could greatly increase the SNR. As shown in Fig. 7, if the traditional IOS processing method was used, the SNR of IOSs were inferior to those with the adaptive IOS processing algorithm applied. In addition, the magnitudes of IOSs (positive and negative) were 10 times smaller (Fig. 7b).

### Experimental setup

**Figure 8:**
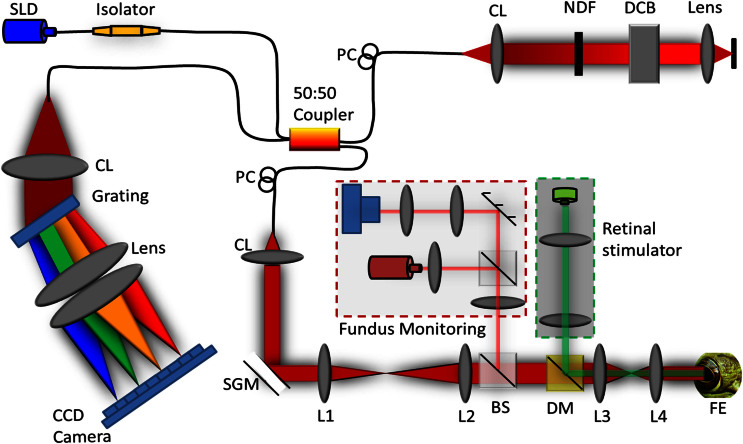
Schematic diagram of the experimental setup. Schematic of the SD-OCT system configured with the retinal stimulator (indicated within green dashed line) and fundus camera (indicated within red dashed line) for *in vivo* functional IOS imaging of the retina. SLD: superluminescent diode, PC: polarization controller, CL: collimation lens, NDF: neutral density filter, DCB: dispersion compensating block, SGM: scanning galvanometer mirror, BS: beam splitter, DM: dichroic mirror, FE: frog eye, CCD: charge-coupled device, L1–L4: lens. Focal lengths of lenses L1, L2, L3 and L4 were 75 mm, 100 mm, 35 mm, and 25 mm, respectively.

A schematic diagram of the custom-designed SD-OCT system with fundus camera and retinal stimulator is illustrated in Fig. 8. The light source used in the OCT system was a broadband superluminescent diode (SLD; Superlum Ltd., Ireland) with a center wavelength of 846 nm and full width at half maximum (FWHM, Δλ) of 104 nm. The theoretical axial resolution of the OCT system was 3.04 μm. The axial resolution experimentally measured was approximately 4 μm in air, corresponding to 3 μm in tissues^48^ (the refractive index of the retina is approximately 1.35). The size of the light beam on the cornea was 2.1 mm and the estimated lateral resolution was around 3 μm. For the fundus imaging subsystem, the light source was a NIR light-emitting diode (LED). A multi-mode optical fiber was used to couple the light into the lens. The optical power entering the pupil was measured to be ~1.5 mW, which met safety requirements. The spectrometer consisted of an IR achromatic f = 150 mm doublet lens (Thorlabs, Inc., USA) as the collimator, a 1200 line/mm transmission grating (Wasatch Photonics, Inc., USA) and rapid rectilinear lenses with two 300 mm achromatic doublet lenses (Thorlabs, Inc., USA). The detector was a linear charge-coupled device (CCD) camera (EV71YEM4CL2014-BA9 OCT/Spectrometer versions, E2V, NY, USA) with 2,048 pixels and 14 μm × 28 μm pixel sizes. The A-line (depth scan) rate of the OCT system was set at 20 kHz for a 100 Hz frame rate and 32 kHz for a 500 Hz frame rate. The visible light stimulus was produced by a single-mode fiber coupled 532 nm DPSS laser module (FC-532-020-SM-APC-1-1-ST; RGBLase LLC, Fremont, CA). The estimated stimulus flash intensity was 5 × 10^5^ photons/μm^2^/ms (5 × 10^6^ photons/μm^2^ for 10 ms) at the retina.

## Supplementary Material

Supplementary Information Video legend

Supplementary Information Dynamic IOSs with a 10 ms flash stimulus for a2

Supplementary Information Dynamic IOSs with a 10 ms flash stimulus for a3

Supplementary Information Dynamic IOSs with a 10 ms flash stimulus up to 7 s recording time

Supplementary Information Dynamic IOSs with a 500 ms stimulus up to 7 s recording time

Supplementary Information Transient IOSs in the dark condition

Supplementary Information transient IOSs in the light condition

Supplementary Information Two OCT images before and after stimulation showing transient photoreceptor displacement

Supplementary Information in vivo OCT retinal images before image registration

Supplementary Information in vivo OCT retinal images after image registration
