# Supplementary material for: Functional Optical Coherence Tomography Enables In Vivo Physiological Assessment of Retinal Rod and Cone Photoreceptors
Source: Sci Rep. 2015 Apr 22;5:9595. doi: 10.1038/srep09595 (PMC4894434; doi:10.1038/srep09595)
Supplement: Supplementary Information — Video legend [file srep09595-s1.pdf]

Functional Optical Coherence Tomography Enables *In Vivo* Physiological Assessment of Retinal Rod and Cone Photoreceptors

Qiuxiang Zhang[1], Rongwen Lu[1], Benquan Wang[1], Jeffrey D. Messinger[2], Christine A. Curcio[2], and Xincheng Yao[1,2,3]*

[1]Department of Biomedical Engineering, University of Alabama at Birmingham, Birmingham, AL 35294

[2]Department of Ophthalmology, University of Alabama at Birmingham, Birmingham, AL 35294

[3]Department of Bioengineering, University of Illinois at Chicago, Chicago, IL 60607

[*]Corresponding author: xcy@uic.edu

Video Legend:

Video 1: Dynamic IOSs with a 10 ms flash stimulus for a2

Video 2: Dynamic IOSs with a 10 ms flash stimulus for a3

Video 3: Dynamic IOSs with a 10 ms flash stimulus up to 7 s recording time

Video 4: Dynamic IOSs with a 500 ms stimulus up to 7 s recording time

Video 5: Transient IOSs in the dark condition

Video 6: Transient IOSs in the light condition

Video 7: Two OCT images before and after stimulation showing transient photoreceptor displacement

Video 8: In vivo OCT retinal images before image registration

Video 9: In vivo OCT retinal images after image registration
